# The Relationship Between Physical Activity, Social Support, and Life Satisfaction Among Female College Students: A Variable- and Person-Centered Analysis

**DOI:** 10.3390/bs16061040

**Published:** 2026-06-22

**Authors:** Yan Liu, Wenying Huang, Wen Zhang, Chang Hu

**Affiliations:** 1 School of Physical Education, Jiangxi Normal University, Nanchang 330022, China; 202541900075@jxnu.edu.cn (Y.L.); zhangwen@jxnu.edu.cn (W.Z.); 2 School of Physical Education, Yunnan University, Kunming 650500, China

**Keywords:** physical activity, social support, life satisfaction, female college students, variable-centered analysis, person-centered analysis, latent profile analysis

## Abstract

Life satisfaction (LS) is an important indicator of subjective well-being among college students. However, relatively few studies have integrated variable-centered and person-centered approaches to examine the associations among physical activity (PA), social support (SS), and LS in female college students. This cross-sectional study surveyed 2097 female college students from 11 universities in Jiangxi Province, China. PA, SS, and LS were assessed using self-report questionnaires. A mediation model was used to examine whether SS statistically mediated the association between PA and LS after controlling for education level and place of origin. Latent profile analysis was then conducted using six LS items, and the BCH method was used to compare PA and SS across profiles. The results showed that PA was positively associated with SS and LS, and SS was positively associated with LS. The indirect association between PA and LS through SS was statistically significant, suggesting a partial statistical mediation pattern. Latent profile analysis identified three level-based LS profiles: low-, medium-, and high-LS profiles. PA and SS increased progressively across these profiles, with the highest levels in the high-LS profile and the lowest levels in the low-LS profile. These findings suggest that PA, SS, and LS are closely interrelated and that meaningful quantitative heterogeneity exists in LS among female college students. Given the cross-sectional design and convenience sampling, the findings should be interpreted as statistical associations rather than causal effects.

## 1. Introduction

Life satisfaction (LS), as a key indicator of subjective well-being and psychological adjustment, has attracted increasing scholarly attention in college student research. LS refers to individuals’ cognitive evaluation of their overall quality of life in terms of self-determined standards and is widely considered a core component of well-being (Diener et al., 2018). During the university years, students experience multiple developmental tasks and contextual demands, including academic workload, interpersonal adaptation, and future career planning, all of which may be associated with their overall life evaluation. Prior research suggests that college students’ LS is generally moderate, with female students often reporting slightly lower LS than male students (W. Gao et al., 2020; Joshanloo & Jovanović, 2020). Surveys also indicate that only about 30–40% of female college students fall within a high LS range, whereas a substantial proportion report moderate or lower LS (Rogowska et al., 2021). For female students, LS may be closely related to academic experiences, health status, interpersonal relationships, body-related experiences, and perceived social resources, making this group a worthy focus in university well-being research (Xu et al., 2025). The present study focused on female college students from 11 universities in Jiangxi Province, China. This population was selected because female students’ LS may be closely related to physical activity participation, perceived interpersonal support, and campus adaptation. Jiangxi Province provided an accessible and feasible regional setting for recruiting a relatively large multi-university sample. Although this regional sample is not nationally representative, it included students from both urban and rural backgrounds, allowing the study to examine PA, SS, and LS within a provincial higher education context. Therefore, the findings may offer preliminary evidence for understanding female students’ well-being in regional Chinese universities and provide a basis for future studies with broader and more representative samples.

### 1.1. Physical Activity and Life Satisfaction

Physical activity (PA), as a positive lifestyle behavior, has been widely associated with both physical and mental health (Biddle & Asare, 2011; Hu et al., 2026a, 2026c). From the perspective of health psychology, regular engagement in PA may be related to lower stress, better emotion regulation, greater self-efficacy, and stronger perceived control, all of which are theoretically relevant to happiness and life satisfaction (Chen et al., 2026; W. Huang et al., 2025). Empirical studies among college students have also shown that PA is positively associated with LS, with more physically active students tending to report better emotional stability, psychological resilience, and social participation (Y.-H. Li et al., 2025; Yu et al., 2025; W. Zhang et al., 2025). Among female college students, sustained and appropriate PA may be particularly relevant to more positive life evaluations because it is often associated with lower negative affect, such as anxiety and depressive symptoms, as well as more adaptive stress experiences, body image, and self-acceptance (Hu et al., 2025; B. Huang et al., 2026; W. Zhang et al., 2026). In addition, PA may also be embedded in social contexts. Compared with solitary exercise, group-based PA, such as physical education classes, sports clubs, and team sports, may provide more opportunities for interpersonal contact, belongingness, and social connectedness, which are closely related to richer social experiences and support resources (Eime et al., 2013; Hu et al., 2026b). Therefore, PA should not be understood only as a health-related behavior, but also as a potential behavioral context statistically associated with social engagement and positive psychological resources on campus.

### 1.2. The Mediating Role of Social Support

Given the cross-sectional design of this study, social support (SS) is examined as a statistical mediator rather than as evidence of a causal mechanism. SS refers to the emotional care, understanding, and resource support that individuals perceive from their social relationships and represents an important interpersonal resource associated with psychological health and social adaptation (Mukta & Akter, 2025). For female college students, SS may be particularly relevant to LS, because perceived support from family, peers, teachers, and the broader campus environment is closely related to emotional comfort, coping resources, and positive evaluations of daily life.

The possible indirect association among PA, SS, and LS can be understood through the social ecological model (Wold & Mittelmark, 2018) and the stress-buffering hypothesis (Wen et al., 2022). These perspectives emphasize that students’ psychological adjustment is embedded in multiple interpersonal contexts, including family, peer, and school environments (X. Zhang et al., 2021). Within this framework, PA may be related to SS because some forms of physical activity, such as extracurricular activities, sports clubs, physical education classes, and team activities, occur in socially interactive settings. These contexts may be associated with broader peer contact, stronger interpersonal connections, and greater opportunities to receive encouragement, recognition, and practical assistance from others (Belcher et al., 2021; Ji & Zheng, 2021).

SS is also consistently associated with LS. Students who perceive greater support may report greater emotional comfort, informational guidance, and practical assistance when facing academic, interpersonal, and career-related stressors. Such perceived resources are often linked to lower negative emotions, more adaptive coping, and more favorable overall evaluations of life (Liu et al., 2016; Peng et al., 2024). Prior studies provide converging evidence that PA is positively associated with perceived support from family, peers, and teachers (Y. Huang et al., 2025; Xiong et al., 2025; Zhou et al., 2025) and that SS is positively associated with LS and may buffer the association between stress and adverse mental health outcomes (Acoba, 2024; Battulga et al., 2021; Delfin et al., 2024). Taken together, these findings suggest that SS may help characterize the statistical association between PA and LS among female college students. Therefore, the present study tests whether SS serves as a statistical mediator in the cross-sectional association between PA and LS, while recognizing that temporal ordering and causal pathways cannot be established from the current design.

### 1.3. Latent Profile Analysis

In recent years, person-centered methods have increasingly been applied in college student mental health and well-being research (Xiang et al., 2025; Y. Yuan et al., 2025). Existing LPA studies have provided important evidence that college students are not a homogeneous group. For example, some studies have identified distinct profiles based on psychological adjustment, coping strategies, positive psychological resources, or value-related indicators, and then examined how these profiles differ in outcomes such as mental health, academic adaptation, or LS (Arslan et al., 2024; Freire et al., 2020; Hernández-Torrano & Ibrayeva, 2025; Xie et al., 2023). These studies have helped clarify heterogeneity in students’ broader psychological functioning.

However, the focus of the present study differs from this prior work in two important ways. First, rather than treating LS only as an outcome or as one component of a broader psychological adjustment profile, the present study used the six LS items themselves as profile indicators to identify level-based LS profiles among female college students. This allowed us to examine heterogeneity in LS directly at the item level. Second, previous LPA studies have rarely examined whether PA and SS are unevenly distributed across LS profiles, particularly in female college student samples. Therefore, it remains unclear whether students with different LS profiles also differ systematically in their physical activity participation and perceived social support.

Accordingly, the present study applies LPA to identify latent profiles of LS among female college students and to compare PA and SS across these profiles. This person-centered analysis complements the variable-centered mediation model by addressing a different question: whereas the mediation model examines the average statistical associations among PA, SS, and LS, the LPA examines whether female students show heterogeneous LS profiles and whether PA and SS vary across these profiles. Importantly, the LPA results are interpreted as a model-based classification of LS levels or patterns, rather than as evidence of qualitatively distinct psychological types. In this way, the study aims to provide a more nuanced understanding of the associations among PA, SS, and LS in female college students.

### 1.4. Current Research

Building on the above literature, the present study examined the associations among PA, SS, and LS in female college students from Jiangxi Province, China. Specifically, we first tested whether PA was positively associated with LS and whether SS showed a statistical indirect association between PA and LS under an assumed cross-sectional mediation model. We then used LPA to identify level-based LS profiles and examined whether PA and SS differed across these profiles.

Accordingly, this study proposes the following hypotheses: (**Hypothesis 1**) PA is positively associated with LS among female college students; (**Hypothesis 2**) SS statistically mediates the association between PA and LS under the assumed cross-sectional model; (**Hypothesis 3**) Latent LS profiles can be identified among female college students, and these profiles differ significantly in PA and SS. By testing these hypotheses, this study aims to provide a more nuanced understanding of how PA, SS, and LS are associated in female college students. The findings may also provide preliminary evidence for future campus health promotion and student support strategies that account for heterogeneity in students’ subjective well-being.

## 2. Materials and Methods

### 2.1. Participants

This study employed a cross-sectional survey design, with data collected through an online questionnaire platform. To ensure adequate statistical power for the planned analyses, an a priori power analysis was conducted using G*Power 3.1 for the regression-based mediation model (Kang, 2021). Assuming a medium effect size (f^2^ = 0.15), a significance level of α = 0.05, and a desired statistical power of 0.95, the analysis indicated that a minimum sample size of 119 participants was required. The final valid sample substantially exceeded this threshold, providing sufficient statistical power for the subsequent analyses.

Participants were recruited from 11 universities in Jiangxi Province, China, using convenience sampling. Data collection was conducted centrally through an online questionnaire platform over approximately one month during the fall semester of 2025. The inclusion criteria were as follows: (a) being biologically female; (b) currently enrolled as a university student; and (c) providing voluntary online informed consent. The exclusion criteria were: (a) self-reported history of clinically diagnosed mental disorders; (b) incomplete questionnaire responses; and (c) excessively short response times or abnormal response patterns identified by the platform as invalid. A total of 2280 female college students completed and submitted the questionnaire. After data screening, 183 questionnaires were excluded because of substantial missing data, inattentive responding, or abnormal response patterns, resulting in a final valid sample of 2097 participants and a valid questionnaire rate of 91.97%.

Before completing the survey, all participants read and confirmed an online informed consent statement. The statement explained that participation was entirely voluntary, that the survey was anonymous, and that all data would be used solely for research purposes and kept strictly confidential. Participants were also informed that they could withdraw from the study at any time without any negative consequences. The mean age of the participants was 20.44 years (SD = 1.99). Most participants were undergraduates (90.70%), followed by master’s students (8.20%) and doctoral students (1.10%).

Regarding residence background, 1224 participants (58.40%) were from urban areas, whereas 873 participants (41.60%) were from rural areas. As compensation for their time and effort, participants who completed and submitted a valid questionnaire received 5 RMB (approximately 0.7 USD) via electronic transfer. All procedures involving human participants were reviewed and approved by the relevant institutional ethics committee, and informed consent was obtained from all participants before participation.

### 2.2. Measurements

#### 2.2.1. Physical Activity Rating Scale

Physical activity was assessed using the Physical Activity Rating Scale developed by Liang (1994). The scale includes three items assessing exercise intensity, exercise duration, and exercise frequency. Exercise intensity and frequency are scored from 1 to 5, whereas exercise duration has five response categories coded from 0 to 4. The total PA score was calculated using the standard formula: PA = intensity × duration × frequency, yielding a theoretical score range from 0 to 100. Total scores were then classified into three levels: low (0–19), moderate (20–42), and high (43+), with higher scores indicating greater physical activity. This instrument has been widely used among Chinese college students and has demonstrated good applicability (Jiang & Wang, 2025; S. Yuan & You, 2022). In the present study, reliability analysis showed a Cronbach’s α of 0.752, indicating acceptable internal consistency.

#### 2.2.2. Social Support Scale

SS was measured using the 12-item Social Support Scale, revised by Blumenthal (Blumenthal et al., 1987), which assesses perceived support from family, friends, and other sources. Items were rated on a 5-point Likert scale ranging from 1 (strongly disagree) to 5 (strongly agree). For analysis, SS was operationalized as the mean score across the 12 items (range 1–5), with higher scores indicating greater perceived social support. This scale has been widely applied in Chinese populations and has demonstrated good applicability (Tang et al., 2021; G. Zhao et al., 2022). In the present study, reliability analysis using SPSS yielded a Cronbach’s α of 0.843 for the total scale, indicating high internal consistency.

#### 2.2.3. College Students’ Life Satisfaction Scale

LS was assessed using the College Students’ Life Satisfaction Scale (Y. Wang & Shi, 2003). The scale consists of six items assessing satisfaction across major life domains and overall life satisfaction. Items were rated on a 7-point Likert scale (1 = strongly disagree to 7 = strongly agree). In the present study, LS was computed as the mean score across the six items (range 1–7), with higher scores indicating higher LS. Reliability analysis showed a Cronbach’s α of 0.833. The CSLSS has been widely used in Chinese samples and has demonstrated good applicability (Xia et al., 2022; Xie et al., 2023), making it suitable for assessing LS in the present research context.

### 2.3. Data Analysis

Data were analyzed using SPSS 26.0 and Mplus 8.3, with a significance level of *p* < 0.05 (Fernández et al., 2020). First, descriptive statistics were computed for all key variables, and Pearson correlation analyses were conducted to examine bivariate associations among PA, SS, and LS. The distribution of key variables was evaluated using skewness, kurtosis, histograms, and Q-Q plots. Because of the large sample size, formal normality tests were not used as the sole criterion for judging normality. The observed PA range was also checked against the theoretical scoring range to confirm correct scoring and identify potential extreme values.

Second, a variable-centered mediation model was tested using the PROCESS macro for SPSS (Hayes, 2018), Model 4. Because the data were cross-sectional, the mediation model was used to test statistical associations rather than causal processes, and all interpretations were limited to the statistical level. PA was specified as the independent variable (X), SS as the mediator (M), and LS as the dependent variable (Y). Education level and place of origin were entered as basic demographic covariates rather than as comprehensive adjustments for all potential confounding factors. Education level was coded as 1 = undergraduate student, 2 = master’s student, and 3 = doctoral student; place of origin was coded as 0 = rural area and 1 = urban area. These covariates were selected because prior research has suggested that demographic and background characteristics may be associated with PA and LS among college students. The indirect effect was evaluated using a bootstrap procedure with 5000 resamples, and bias-corrected 95% confidence intervals (CIs) were computed. An indirect effect was considered statistically significant when the corresponding CI did not include zero.

Third, to examine heterogeneity in LS, a person-centered latent profile analysis (LPA) was performed in Mplus 8.3 using the six CSLSS items (sh1–sh6) as continuous indicators (Wei et al., 2021). Models with increasing numbers of profiles were estimated and compared using the Akaike information criterion (AIC), the Bayesian information criterion (BIC), and the sample-size-adjusted BIC, with lower values indicating better fit. The Lo–Mendell–Rubin adjusted likelihood ratio test (LMR-LRT) and the bootstrap likelihood ratio test (BLRT) were used to compare the k-profile model with the k − 1-profile model. Classification accuracy was assessed using entropy. Based on overall model fit, parsimony, and interpretability, a three-profile solution was selected. The three profiles were labeled the low-LS, medium-LS, and high-LS groups.

Finally, after determining the optimal profile, the BCH method was used to compare PA and SS across the three LS profiles, accounting for classification uncertainty. Wald chi-square tests were used to evaluate overall profile differences, followed by pairwise comparisons when significant differences were observed.

## 3. Results

### 3.1. Common Method Bias

Because all variables were collected from the same respondents using self-report questionnaires, common method bias was examined using both Harman’s single-factor test and a single-factor confirmatory factor analysis. First, Harman’s single-factor test indicated that four factors had eigenvalues greater than 1, and the first unrotated factor accounted for 30.60% of the total variance, which was below the commonly used 40% threshold (Podsakoff et al., 2003). This result suggested that no single factor dominated the covariance among the measured variables. Second, a single-factor confirmatory factor analysis was conducted by loading all observed items onto one latent factor. The results indicated that the single-factor model showed poor fit to the data, χ^2^/df = 18.97, CFI = 0.73, TLI = 0.71, and RMSEA = 0.09. These findings suggest that a single common factor could not adequately account for the covariance among the study variables. Taken together, although common method variance cannot be completely ruled out due to the cross-sectional self-report design, the results indicate that common method bias was unlikely to be a major threat to the validity of the findings.

### 3.2. Correlational Analysis

Descriptive statistics, observed score ranges, skewness, kurtosis, and Pearson correlations among PA, SS, and LS were computed (Table 1). The observed score ranges of PA, SS, and LS were all within their theoretical scoring ranges, indicating no obvious scoring errors. PA scores showed relatively large dispersion, with an observed range of 0–100, a mean of 26.59, and a standard deviation of 30.20. The skewness and kurtosis values for PA were 0.97 and −0.41, respectively, suggesting a moderately positively skewed distribution that is not severely non-normal. For SS, the skewness and kurtosis values were −0.14 and −0.54, respectively, and for LS, they were 0.06 and −0.35, respectively. These values indicated that SS and LS were approximately symmetrically distributed, and that none of the key variables showed severe deviations from normality. Visual inspection of the histograms and Q-Q plots further suggested that the distributions were acceptable for subsequent parametric analyses.

Pearson correlation analyses showed that PA was positively correlated with SS (*r* = 0.486, *p* < 0.01) and LS (*r* = 0.332, *p* < 0.01). SS was also positively correlated with LS (*r* = 0.501, *p* < 0.01).

### 3.3. Mediation Analysis

A statistical mediation model was tested using the PROCESS macro (Model 4) to examine the indirect association between PA and LS through SS under the assumed cross-sectional model. Education level and place of origin were included as covariates. Education level was entered as an ordinal covariate and coded as 1 = undergraduate student, 2 = master’s student, and 3 = doctoral student. Place of origin was coded as 0 = rural area and 1 = urban area. Multicollinearity was assessed using variance inflation factors (VIFs), and all VIF values were below the conventional threshold of 5, indicating no serious multicollinearity.

As shown in Table 2, PA was positively associated with SS (*β* = 0.482, *t* = 25.176, *p* < 0.001). In the model predicting LS, both PA, *β* = 0.117, *t* = 5.656, *p* < 0.001, and SS, *β* = 0.442, *t* = 21.352, *p* < 0.001, were positively associated with LS. Education level was negatively associated with LS, *β* = −0.667, *t* = −12.523, *p* < 0.001. This coefficient is a standardized regression coefficient. Given the ordinal coding of education level, the negative coefficient indicates that a higher education level was associated with lower LS in the present sample. However, because the sample was predominantly composed of undergraduate students, with relatively small proportions of master’s and doctoral students, this coefficient should be interpreted cautiously.

**Figure 1:**
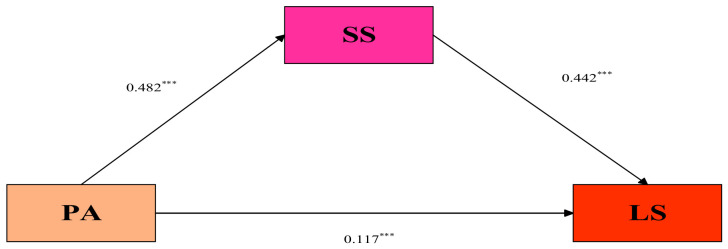
Mediation effect diagram. Path coefficients are from cross-sectional data and reflect statistical associations, not causal effects. *** *p* < 0.001.

As shown in Table 3 and Figure 1, the total association between PA and LS was 0.330. The direct association was 0.117, and the statistical indirect association through SS was 0.213. The bootstrap 95% CI for the statistical indirect association did not include zero, BootLLCI = 0.186, and BootULCI = 0.243, indicating that SS statistically accounted for part of the association between PA and LS under the assumed cross-sectional model.

The statistical indirect association accounted for approximately 65% of the total association, whereas the direct association accounted for approximately 35%. These findings indicate a statistical mediation pattern under the assumed cross-sectional model, in which SS was statistically associated with part of the relationship between PA and LS. Given the cross-sectional design, this result should be interpreted as a statistical indirect association rather than evidence of a causal pathway or temporal mechanism.

### 3.4. Latent Profile Analysis

Consistent with Hypothesis 3, LPA was conducted using the six LS items to examine whether female college students could be classified into empirically derived profiles based on their item-level LS responses. Models specifying one to five profiles were estimated and compared. As shown in Table 4, AIC, BIC, and aBIC decreased as the number of profiles increased, suggesting improved relative model fit. In determining the optimal solution, model parsimony, interpretability, entropy, profile size, and the LMR-LRT and BLRT results were considered together.

The three-profile solution was selected for several reasons. First, compared with the two-profile model, the three-profile model showed a substantial decrease in AIC, BIC, and aBIC, indicating improved relative model fit. Second, although the four- and five-profile models produced further decreases in information criteria, this pattern is common in mixture modeling as model complexity increases. Therefore, the optimal solution should not be determined solely by the lowest information criteria. Instead, parsimony, classification accuracy, profile size, and substantive interpretability should also be considered.

Third, the three-profile solution had the highest entropy among the multi-profile models, at 0.925, compared with 0.924 for the four-profile model and 0.921 for the five-profile model. This indicates that the three-profile model provided slightly better classification accuracy. Fourth, the three-profile model yielded relatively stable and interpretable profile sizes, with 15.02%, 64.00%, and 20.98% of participants classified into the three profiles. In contrast, the four- and five-profile solutions introduced additional smaller profiles without providing clearly meaningful new response patterns. These additional profiles appeared to split existing level-based groups further rather than reveal substantively distinct LS patterns.

After the three-profile solution was selected, the average posterior probabilities were further examined to evaluate the classification quality of the latent profiles. As shown in Table 5, the diagonal values were high for all three profiles, with probabilities of 0.977 for the low-LS profile, 0.969 for the medium-LS profile, and 0.956 for the high-LS profile. These values indicate that participants assigned to each profile had a high probability of belonging to their corresponding profile. The off-diagonal probabilities were relatively low, suggesting limited overlap in classification among the three profiles. Overall, the average posterior probabilities supported good classification accuracy for the three-profile solution.

**Figure 2:**
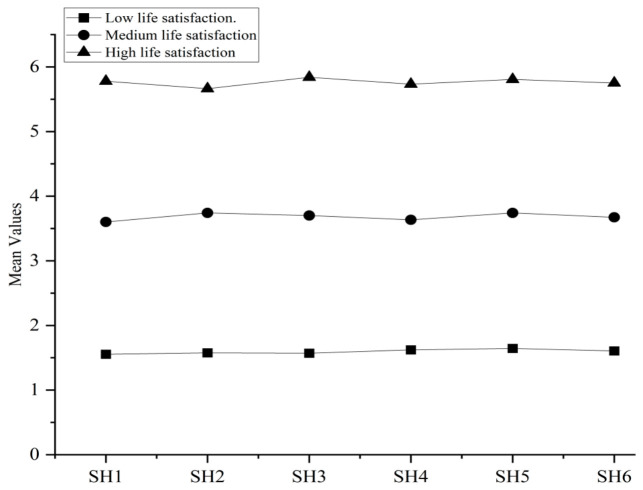
Scores and distribution of the three latent profiles of life satisfaction. Note: SH1–SH6 represent the six items of the life satisfaction scale.

Based on the mean scores across the six LS items (SH1–SH6), the three profiles were labeled as low-, medium-, and high-LS profiles. Specifically, the low-LS profile showed the lowest scores across all six items, indicating generally low LS; the medium-LS profile displayed mid-range scores with a relatively flat pattern across items, reflecting an overall moderate level of LS; and the high-LS profile exhibited the highest scores across all items, reflecting consistently high satisfaction across life domains (Figure 2). Overall, the three profiles demonstrated similar patterns across items but differed clearly in overall level, suggesting that the identified profiles mainly reflected quantitative differences in LS rather than qualitatively distinct response patterns across LS domains.

It should be noted that these profiles should not be interpreted as distinct psychological types. Rather, they represent empirically derived, level-based LS profiles identified from the joint distribution of the six LS items. Compared with arbitrarily dividing the total LS score into low, medium, and high groups, LPA provides a model-based classification approach that uses item-level information, evaluates classification accuracy, and allows subsequent comparisons across profiles while accounting for classification uncertainty. Therefore, the LPA results provide evidence of quantitative heterogeneity in LS within this sample and offer a basis for examining whether PA and SS differ systematically across level-based LS profiles.

### 3.5. Effects of Latent Profile Membership

To further examine profile differences, the BCH method was used to compare PA and SS across the three LS profiles while accounting for classification uncertainty. As shown in Table 6, significant differences were found for both PA (Wald *χ*^2^ = 414.074, *p* < 0.001) and SS (Wald *χ*^2^ = 938.987, *p* < 0.001).

Pairwise comparisons showed a clear gradient pattern. The high-LS profile reported the highest PA and SS, followed by the medium-LS profile, whereas the low-LS profile reported the lowest levels. Specifically, PA increased from 8.526 in the low-LS profile to 25.095 in the medium-LS profile and 43.762 in the high-LS profile. SS showed a similar pattern, increasing from 2.033 to 3.223 and 3.649 across the three profiles. These results indicate that PA and SS differed systematically across LS profiles, providing support for Hypothesis 3.

## 4. Discussion

This study used variable-centered and person-centered approaches to examine associations among PA, SS, and LS in female college students. The variable-centered results showed that PA was positively associated with LS, and SS statistically accounted for part of this association under the hypothesized mediation model. The person-centered results identified three level-based LS profiles that differed systematically in PA and SS. Together, these findings clarify the overall associations among PA, SS, and LS and show quantitative heterogeneity in LS within this sample. The negative association between education level and LS should be interpreted cautiously. Graduate students may experience greater academic, research, career, and graduation-related pressures, which may be associated with lower LS (Guan et al., 2025). However, because the sample was predominantly composed of undergraduate students and the numbers of master’s and doctoral students were relatively small, this finding requires further examination in more balanced samples.

The study found a significant positive association between PA and LS among female college students, supporting Hypothesis 1 and aligning with previous findings (Kotarska et al., 2021; Pedišić et al., 2015). This association may be understood from several theoretical perspectives. According to the self-efficacy theory, students who engage in more PA may report higher self-efficacy and perceived control (Han et al., 2022), which are often linked to more adaptive coping and more positive evaluations of life (Raimondi et al., 2025). From the perspective of cognition and emotion regulation, PA is also associated with more positive affective states, reduced negative emotional experiences, and greater psychological resources, all of which may be related to higher LS (Archer et al., 2014; Z. Zhang et al., 2022). In addition, some forms of PA, especially group-based or campus-based activities, may provide opportunities for interpersonal contact and supportive social experiences, which may also be associated with more favorable life evaluations (Ye et al., 2025; X. Zhao et al., 2024). However, given the cross-sectional design, these interpretations should be understood as theoretically plausible explanations of the observed association rather than evidence of causal effects (H. Wang et al., 2025).

Further analyses showed that SS statistically accounted for part of the association between PA and LS, supporting Hypothesis 2 at the level of statistical mediation. This finding is consistent with previous evidence suggesting that SS is closely related to both PA and subjective well-being (C.-C. Lo et al., 2025; Jiang et al., 2025). One possible explanation is that students with higher PA may also have more opportunities for peer interaction, encouragement, and recognition in campus activity contexts, particularly in team sports or sports clubs (Patterson et al., 2025). At the same time, students with higher perceived SS may experience stronger feelings of understanding, acceptance, belonging, and self-worth, which are often associated with higher LS (K. Lee, 2020).

This interpretation is consistent with the stress-buffering perspective of SS (J. Lo, 2019) and the conservation of resources theory (Holmgreen et al., 2017), which emphasize the role of social resources in adjustment and well-being. Nevertheless, because all variables were measured at the same time point, the mediation result should be interpreted as a statistical decomposition of associations under an assumed model, rather than evidence that SS is a causal pathway linking PA to LS. Future longitudinal or experimental studies are needed to clarify the temporal ordering among PA, SS, and LS (Chang et al., 2023; Hanimoğlu, 2025). From a practical perspective, the present findings suggest that universities may simultaneously promote PA and strengthen supportive social environments. For example, peer-led activity groups, inclusive sports clubs, and supportive campus climates may help create contexts in which students can engage in PA while also experiencing greater interpersonal support. However, these recommendations remain tentative and should be further tested in longitudinal and intervention research (Chang et al., 2023).

Building on the variable-centered analyses, this study further adopted a person-centered approach to examine heterogeneity in LS. The LPA identified three level-based LS profiles, namely low, medium, and high LS, supporting Hypothesis 3. These profiles mainly differed in overall level rather than in qualitatively distinct item-response patterns (Aydin et al., 2024; Hernández-Torrano et al., 2020). Therefore, they should not be interpreted as distinct psychological types, but rather as empirically derived profiles reflecting quantitative heterogeneity in LS. Compared with simple grouping based on total-score cutoffs, LPA provides a model-based classification using item-level information and allows for evaluation of classification quality. In addition, the subsequent BCH analysis allowed PA and SS to be compared across profiles while accounting for classification uncertainty (Wu et al., 2020).

The BCH results showed significant differences in PA and SS across the three LS profiles. Specifically, students in the high-LS profile reported the highest levels of PA and SS, those in the medium-LS profile showed intermediate levels, and those in the low-LS profile reported the lowest levels (H. Li & Hao, 2025; Moreno-Murcia et al., 2017). This gradient pattern is consistent with the variable-centered results, suggesting that higher PA and stronger perceived SS tend to co-occur with higher LS (Z. Gao et al., 2024; Hou et al., 2025; X. Wang et al., 2024). These findings provide converging evidence from both variable-centered and person-centered perspectives, while also indicating that LS heterogeneity in this sample was primarily quantitative in nature.

From a social ecological perspective, the observed profile differences may be related to multiple contextual and interpersonal factors, including family background, campus exercise resources, peer relationships, and broader social environments (Eriksson et al., 2025; Feng & Li, 2025). Prior research has shown that college students’ PA and SS are associated with environmental and resource-related conditions, such as sports facilities, campus exercise culture, peer support, and opportunities for social participation (J. Zhang & Phee, 2025; Van Luchene & Delens, 2021). Female students with lower LS may have fewer opportunities for PA participation, weaker perceived support, or less positive social feedback in daily campus life (Brown et al., 2024; Burton et al., 2021). However, because these contextual factors were not directly measured in the present study, this explanation should be regarded as tentative. Future research should include more detailed measures of PA context, sources of SS, and campus environmental factors to clarify why students with different LS levels also differ in PA and SS (Alsubaie et al., 2019).

Overall, the person-centered findings complement the mediation analysis by showing that PA and SS are not only associated with LS at the variable level (J. Lee et al., 2021) but also differ systematically across level-based LS profiles. However, the profiles should be understood as low-, medium-, and high-LS groups identified through a model-based approach, rather than as qualitatively distinct subgroups. This more cautious interpretation provides a clearer basis for future studies to examine whether profile-informed support strategies are useful for students with different levels of LS.

### Implications and Limitations

The present study has several strengths. In the present sample, PA and SS were positively associated with LS, and SS showed a statistically significant indirect association between PA and LS under the assumed cross-sectional model. These results do not demonstrate that increasing PA or SS would directly improve LS. Nevertheless, they are consistent with existing evidence suggesting that physical activity participation and supportive interpersonal environments are both relevant to student well-being. Therefore, universities may consider these findings as preliminary evidence when developing broader health promotion and student support strategies. For example, campus initiatives could aim to provide more accessible physical activity opportunities while also fostering supportive peer, teacher, and dormitory environments. Such strategies may be especially relevant in programs that seek to promote students’ general well-being, but their effectiveness should be evaluated in future longitudinal or intervention studies.

The person-centered findings also provide tentative information for understanding heterogeneity in female college students’ LS. Students in the low-LS profile reported lower levels of PA and SS, whereas those in the high-LS profile reported higher levels of both variables. This pattern suggests that students with lower LS may also have fewer behavioral and interpersonal resources, although the present study cannot determine the direction or cause of these associations. Accordingly, profile-informed support should be viewed as a possible direction for future research and practice rather than as a direct implication confirmed by the current data. Future studies could examine whether students with different LS profiles respond differently to campus-based physical activity programs, peer support activities, or other well-being initiatives.

Several limitations should be noted. First, the cross-sectional design prevents causal inference. Although the mediation model assumed that PA was indirectly associated with LS via SS, all variables were measured at a single time point using self-report questionnaires. Therefore, the indirect effect should be interpreted as a statistical decomposition of associations under an assumed model rather than evidence of a causal mechanism or temporal process. Alternative directional explanations, such as SS being associated with both PA and LS, or LS being associated with both PA and SS, cannot be ruled out. Unmeasured common causes, such as personality traits, family environment, or prior mental health status, may also have influenced the observed associations. Future longitudinal, experimental, or cross-lagged studies are needed to clarify the temporal ordering among PA, SS, and LS.

Second, the covariates included in the mediation model were limited. This study controlled only for education level and place of birth. However, LS among college students may also be associated with other important factors, such as physical health status, sleep quality, depressive symptoms, anxiety, academic stress, BMI, family socioeconomic status, and personality traits. Because these variables were not fully measured in the present study, residual confounding may exist. Therefore, the present findings should not be interpreted as being independent of all major confounding factors.

Third, the sampling strategy limits the generalizability of the findings. Participants were recruited through convenience sampling from 11 universities in Jiangxi Province. Although the sample size was relatively large, the sample may not fully represent female college students in other regions of China or in different types of institutions. In addition, detailed information on institution type, academic discipline, year level, and socioeconomic background was not fully available. Future studies should recruit more diverse and representative samples across regions, institution types, majors, and grade levels, and should collect richer demographic and contextual information.

Fourth, PA, SS, and LS were measured mainly by self-report questionnaires, which may be affected by recall bias, social desirability, and shared-method variance. Although common method bias was assessed statistically, it cannot be completely ruled out. Future research should incorporate objective and multi-source measures, such as wearable PA monitoring, peer or teacher ratings of SS, and repeated assessments of LS.

Fifth, the measurement of PA did not distinguish between solitary and socially embedded forms of activity. The theoretical explanation that PA may be associated with higher SS through interpersonal interaction applies most directly to group-based or team-based PA. Therefore, the observed indirect association via SS may reflect, but does not isolate, the contribution of socially embedded PA. Future studies should include context-specific measures of PA, such as individual exercise, group exercise, sports club participation, and team sports.

Finally, LPA involves classification uncertainty. Although the three-profile solution was selected based on model fit, parsimony, and interpretability, the profiles should be replicated in independent samples. Future research may also use methods that account for classification error and examine whether the identified profiles remain stable over time. In addition, the proportion of the indirect effect should be interpreted cautiously. In cross-sectional data, such proportions are descriptive and do not imply causal decomposition.

## 5. Conclusions

This study integrated variable-centered and person-centered approaches to examine the associations among PA, SS, and LS among female college students. The results showed that both PA and SS were positively associated with LS, and that SS statistically accounted for part of the association between PA and LS in the hypothesized cross-sectional mediation model. The LPA further identified three level-based LS profiles, namely low-, medium-, and high-LS profiles, with progressively higher PA and SS observed across higher LS profiles. These findings suggest that PA, SS, and LS are closely interrelated and that meaningful heterogeneity exists in LS among female college students. From a practical perspective, universities may consider providing accessible PA opportunities, strengthening supportive campus environments, and offering profile-informed support for students with lower LS. However, because this study used a cross-sectional design and convenience sampling, the findings should be interpreted as statistical associations rather than causal effects and should be further examined in longitudinal and intervention studies.

## Figures and Tables

**Table 1:** Descriptive statistics and correlations (*n* = 2097).

Variable	M	SD	Observed Range	Skewness	Kurtosis	PA	SS	LS
PA	26.59	30.20	0–100	0.97	−0.41	1		
SS	3.13	0.84	1–5	−0.14	−0.54	0.486 **	1	
LS	3.81	1.35	1–7	0.06	−0.35	0.332 **	0.501 **	1

Note: ** *p* < 0.01.

**Table 2:** Testing the mediation model.

Variable	Social Support		Life Satisfaction	
	*β*	*t*	*β*	*t*
Education level	−0.064	−1.133	−0.667	−12.523 ***
Place of origin	−0.075	−1.928	0.096	2.594 **
Physical activity	0.482	25.176 ***	0.117	5.656 ***
Social support			0.442	21.352 ***
*R* ^2^	0.238		0.318	
*F*	217.322 ***		244.113 ***	

Note: *β* values are standardized regression coefficients. Education level was entered as an ordinal covariate and coded as 1 = undergraduate student, 2 = master’s student, and 3 = doctoral student; place of birth was coded as 0 = rural area and 1 = urban area. The dependent variable is life satisfaction; the independent variable is physical activity; the mediating variable is social support; and the control variables are education level and place of birth. ** *p* < 0.01, *** *p* < 0.001.

**Table 3:** Results of mediation effect analysis.

Path	Effect	BootSE	BootLLCI	BootULCI	Relative Proportion
Total effect	0.33	0.02	0.291	0.369	100%
Direct effect	0.117	0.021	0.076	0.158	35%
Indirect effect	0.213	0.014	0.186	0.243	65%

**Table 4:** Fit statistics for latent profile models based on life satisfaction.

Profiles	AIC	BIC	aBIC	LMR-LRT	BLRT	Entropy	1	2	3	4	5
1	50,845.229	50,913.009	50,874.883	N/A	N/A	1	1				
2	47,957.403	48,064.72	48,004.355	<0.001	<0.001	0.872	73.82	26.18			
3	46,225.918	46,372.773	46,290.169	<0.001	<0.001	0.925	15.02	64.00	20.98		
4	45,794.511	45,980.903	45,876.059	<0.001	<0.001	0.924	14.98	54.98	10.11	19.93	
5	45,407.622	45,633.553	45,506.469	<0.01	<0.001	0.921	15.59	46.64	8.39	9.87	19.51

Note: N/A, not applicable; AIC, Akaike Information Criterion; BIC, Bayesian Information Criterion; aBIC, adjusted Bayesian Information Criterion; LMR-LRT, Lo–Mendell–Rubin Likelihood Ratio; BLRT, Bootstrap Likelihood Ratio Test.

**Table 5:** Average posterior probabilities for the three latent LS profiles.

Assigned Profile	Low-LS Profile	Medium-LS Profile	High-LS Profile
Low-LS profile	0.977	0.023	0
Medium-LS profile	0.009	0.969	0.023
High-LS profile	0	0.044	0.956

**Table 6:** BCH comparisons of PA and SS across latent LS profiles.

Variable	Group	M ± SE	Wald χ^2^	Pairwise Comparison
PA	① Low-LS profile	8.526 ± 0.888	414.074 ***	③ > ② > ①
	② Medium-LS profile	25.095 ± 0.819		
	③ High-LS profile	43.762 ± 1.770		
SS	① Low-LS profile	2.033 ± 0.040	938.987 ***	③ > ② > ①
	② Medium-LS profile	3.223 ± 0.018		
	③ High-LS profile	3.649 ± 0.041		

Note: SE = standard error. Wald *χ*^2^ values were obtained using the BCH procedure. Pairwise comparisons indicate significant differences between all profiles. *** *p* < 0.001.

## Data Availability

The data used in this study are available from the corresponding author upon reasonable request.

## Abbreviations

The following abbreviations are used in this manuscript:

PA	Physical Activity
SS	Social Support
LS	Life Satisfaction
LPA	Latent Profile Analysis
CSLSS	College Students’ Life Satisfaction Scale
AIC	Akaike Information Criterion
BIC	Bayesian Information Criterion
aBIC	Sample Size-Adjusted Bayesian Information Criterion
LMR-LRT	Lo–Mendell–Rubin Likelihood Ratio Test
BLRT	Bootstrap Likelihood Ratio Test
BCH	Bolck–Croon–Hagenaars Method
CI	Confidence Interval
VIF	Variance Inflation Factor
SE	Standard Error
CFI	Comparative Fit Index
TLI	Tucker–Lewis Index
RMSEA	Root Mean Square Error of Approximation
